# The influence of iron status and genetic polymorphisms in the HFE gene on the risk for postoperative complications after bariatric surgery: a prospective cohort study in 1,064 patients

**DOI:** 10.1186/1754-9493-5-1

**Published:** 2011-01-10

**Authors:** Glenn S Gerhard, Ravi Chokshi, Christopher D Still, Peter Benotti, G Craig Wood, Mollie Freedman-Weiss, Cody Rider, Anthony T Petrick

**Affiliations:** 1 Weis Center for Research, Geisinger Clinic, 100 North Academy Avenue, Danville, PA 17822, USA; 2 Dept of Surgery, Geisinger Medical Center, 100 North Academy Avenue, Danville, PA 17822, USA; 3 Geisinger Obesity Research Institute, Geisinger Clinic, 100 North Academy Avenue, Danville, PA 17822, USA; 4 Dept of Surgery, St. Francis Medical Center, Trenton, NJ 08629, USA

## Abstract

**Background:**

Gastric bypass surgery is a highly effective therapy for long-term weight loss in severely obese patients, but carries significant perioperative risks including infection, wound dehiscence, and leaks from staple breakdown. Iron status can affect immune function and wound healing, thus may influence peri-operative complications. Common mutations in the HFE gene, the gene responsible for the iron overload disorder hereditary hemochromatosis, may impact iron status.

**Methods:**

We analyzed 1064 extremely obese Caucasian individuals who underwent open and laparoscopic Roux-n-Y gastric bypass surgery at the Geisinger Clinic. Serum iron, ferritin, transferrin, and iron binding capacity were measured pre-operatively. All patients had intra-operative liver biopsies and were genotyped for the C282Y and H63D mutations in the HFE gene. Associations between surgical complications and serum iron measures, HFE gene status, and liver iron histology were determined.

**Results:**

We found that increased serum iron and transferrin saturation were present in patients with any post-operative complication, and that increased serum ferritin was also increased in patients with major complications. Increased serum transferrin saturation was also associated with wound complications in open RYGB, and transferrin saturation and ferritin with prolonged lengths of stay. The presence of 2 or more HFE mutations was associated with overall complications as well as wound complications in open RYGB. No differences were found in complication rates between those with stainable liver iron and those without.

**Conclusion:**

Serum iron status and HFE genotype may be associated with complications following RYGB surgery in the extremely obese.

## Background

Obesity and metabolic syndrome have been related to abnormalities in iron metabolism and to hepatic iron overload [1-3]. For example, serum ferritin, a commonly used indicator of total body iron stores, increases with BMI [4]. Hepatic iron overload has also been implicated in the pathogenesis of non-alcoholic steatohepatitis (NASH), a common complication of obesity. Roux-en-Y gastric bypass (RYGB), demonstrated to produce long-term maintenance of weight loss in the severely obese, also improves steatosis, necroinflammatory activity, and hepatic fibrosis in patients with morbid obesity and NASH. Although iron deficiency is a long-term complication of RYGB [5], laparoscopic adjustable gastric banding did not normalize serum ferritin levels despite correction of other metabolic abnormalities suggesting that iron overload may persist despite resolution of obesity [6]. The relationship between iron status and short-term perioperative complications of RYGB has not yet been studied.

Iron status is commonly determined through measurement of serum iron, transferrin saturation, and ferritin levels, and through histological assessment of liver biopsies [7,8]. Hepatic iron overload can be attributed to mutations in the HFE gene [9,10], the gene responsible for the iron overload disorder hereditary hemochromatosis, that also cause increases in serum transferrin saturation and ferritin levels. Two mutations in the HFE gene commonly occur, C282Y and H63D [11]. The C282Y mutation is less frequent but when homozygous is more often associated with iron overload. The H63D mutation is more common but far less frequently associated with iron overload. Compound C282Y/H63D heterozygotes are also considered to be at risk for iron overload.

RYGB is a relatively safe surgical procedure for most patients but does carry perioperative risks including infection, dehiscence, leaks from staple breakdown, and ulcers. Iron status can affect immune function by strengthening epithelial barriers and both cellular and humoral immune responses [12]. With the number of RYGB procedures increasing each year, understanding the factors associated with these risks will be important to decrease associated morbidity, decrease costs, and improve outcomes. Therefore, we determined whether the presence of hepatic iron overload, abnormal iron indices, or HFE gene mutations influenced the rate of post-operative complications following RYGB.

## Methods

All bariatric surgical candidates at Geisinger Medical Center were enrolled in the Geisinger Center for Nutrition and Weight Management and were prospectively recruited into a clinical research program on obesity. A cohort of 1064 patients who underwent either open or laparoscopic RYGB with intraoperative wedge liver biopsy between September 2004 to May 2008 were analyzed for this study. Approval for the research was granted by the Institutional Review Board of the Geisinger Clinic and all participants provided written informed consent. The study included only Caucasians of European descent as judged by clinician assessment; those of non-European descent made up only 3% of the study population and were excluded to maintain power in the genetic analyses. Patients undergoing other bariatric procedures or revisional procedures were also excluded. A comprehensive medical history and physical examination was performed during the initial visit. Patients then underwent a minimum of 6 months preoperative assessment and preparation period. During this time a comprehensive set of clinical and laboratory measures (reference ranges) were obtained including serum iron (males 45-160 ug/dL; females 30-160 ug/dL), iron binding capacity (228-428 ug/dL), ferritin (males 30-400 ng/mL; females 13-150 ng/mL), transferrin saturation (15-55%), white blood cell count (WBC: 4.0-10.8 K/uL), red blood cell count (RBC: males 4.50-5.25 M/ul; females 3.85-5.15 M/ul), hemoglobin (males 14.0-16.5 gm/dL; females 12.0-14.5 gm/dL), hematocrit (HCT: males 40-47%; females 36-44.5%), mean cell volume (MCV: males 82-99.5 fL; females 81.5-97.5 fL), mean cell hemoglobin (MCH: 27-34 pg/cell), and mean cell hemoglobin concentration (MCHC: 32-36 gm/dL), platelet count (150-400 K/ul), zinc (60-130 ug/dL), and folic acid (4.2-19.9 ng/mL). Clinical data were extracted from the EpicCare EHR and read into SAS/STAT software (SAS Institute Inc., Cary, NC).

Liver biopsy specimens were formalin fixed and stained with hematoxylin and eosin for routine histology, Masson's trichrome for assessment of fibrosis, and Perls'/Prussian Blue stain to determine iron status. All specimens were read by experienced pathologists using the criteria for NASH of Brunt [13]. DNA was prepared as previously described [14]. Single nucleotide polymorphism (SNP) genotyping was performed on an Applied Biosystems 7500 real-time PCR System (Applied Biosystems, Foster City, CA). Assay reagents for each SNP were obtained from Applied Biosystems (HFE C282Y, rs1800562, C___1085595_10; HFE H63D, rs1799945, C___1085600_10). The reaction was then analyzed using Applied Biosystems Sequence Detection Software. The Institutional Review Board of the Geisinger Clinic approved the research protocol and all participants provided written informed consent.

Patients were followed for a 30-day period to determine length of stay and post-operative complications. Complications [15-19] were categorized as in Table 1.

**Table 1:** Major and minor complications.

Major:	
	Death from any cause
	Reoperations for any cause
	Respiratory including hypoxia, hypercarbia, reintubation, or significant dyspnea
	Bleeding including post-operative blood loss sufficient to require transfusion or reoperation
	Wound complications requiring readmission to the hospital
	Leak including leak from pouch, anastomosis, or excluded stomach
	Thromboembolism including proven deep vein thrombosis or pulmonary embolus
	Gastrointestinal including paralytic ileus, intestinal ischemia, intestinal obstruction or internal hernia
	Acute renal failure including renal failure requiring dialysi
	Other severe problems causing readmission

Minor:	

	Feeding intolerance including delayed discharge because foregut symptoms delayed diet progression
	Cardiac including atrial fibrillation or flutter
	Infection including pneumonia, sepsis, cellulitis, fever, clostridia difficile infection, or wound infection
	Urinary including urinary tract infection, or urinary retention
	Superficial wound complications which did not require or prolong hospitalization
	Minor respiratory problems which did not require or prolong hospitalization
	Stricture including gastro-jejunal anastomotic stricture
	Other minor problems without readmission

Descriptive statistics such as the calculation of means, percentages and confidence intervals were used to describe the study population. Chi-square tests, Fisher's exact tests, and Wilcoxon rank-sum tests were used to compare abnormal iron pathology, serum levels, and gene mutation results between those with and without the primary outcomes (i.e. complications and extended length of stay). Analyses compared the rate of any complication, the rate of major complication, and the rate of minor complications to the no complication group. Wound complications were also evaluated in patients with open RYGB. SAS version 9.1 (Cary, NC) was used for data manipulation and statistical analysis. All tests were two-sided, and p-values < 0.05 were considered significant.

## Results

The mean age of our patients was 46.5 years old of whom 80% were females (Table 2) and all were of Caucasian descent. Hepatic iron staining determined by liver biopsy obtained during RYGB surgery was noted in 17.2% patients (n = 183), while 82.8% (n = 881) of the patients studied had no evidence of increased liver iron. Median serum iron parameters obtained from blood samples obtained prior to surgery fell within the normal range. A total of 11% of the patients studied were either homo- or heterozygous for the C282Y mutation and 27% were either homo- or heterozygous for the H63D mutation (Table 2). Type 2 diabetes and hypertension were present in over one-third of patients, with a 1% incidence of biliary disease (Table 3). Just over 10% were on hormone replacement therapy or were using oral contraceptives. Less than 10% were on oral iron supplementation and most patients were non-smokers and non-drinkers. Approximately two-thirds of patients had hepatic steatosis, one-quarter had hepatic fibrosis, and 2% manifested cirrhosis. The median complete blood counts and folate and zinc levels were normal.

**Table 2:** Demographics and iron parameters of study population (N = 1064).

*Demographic/Iron Parameter*	***Result***
Age (years), Mean (SD)	46.5 (11.0)
Gender	
Male (%)	209 (20%)
Female (%)	855 (80%)
Weight (lbs.), Mean (SD)	
Baseline	307 (62)
Surgery	293 (60)
BMI (kg/m^2^), Mean (SD)	
Baseline	50.2 (8.7)
Surgery	47.9 (8.3)
Hepatic iron staining	183 (17.2%)
Serum iron (ug/dL)*, Median [Q1, Q3]	62 [50, 79]
Ferritin (ng/mL)*, Median [Q1, Q3]	99 [50, 167]
Iron binding capacity (ug/dL)*, Median [Q1, Q3]	316 [287, 349]
Transferrin saturation (%)*, Median [Q1, Q3]	20 [15, 25]
HFE C282 NORMAL	950 (89%)
HFE C282Y HETEROZYGOUS	111 (10%)
HFE C282Y HOMOZYGOUS	3 (<1%)
HFE H63D NORMAL	777 (73%)
HFE H63D HETEROZYGOUS	257 (24%)
HFE H63D HOMOZYGOUS	30 (3%)

*Serum iron, ferritin, iron binding capacity, and transferring saturation were available for n = 894, n = 900, n = 895, and n = 891 subjects, respectively.

**Table 3:** Characteristics of the study population (N = 1064).

Characteristic	Result
Diabetes (%)	373 (35%)
Hypertension (%)	451 (42%)
Biliary disease (%)	15 (1%)
Contraceptive or estrogen use (%)	116 (11%)
Iron supplementation (%)	80 (8%)
Tobacco Use (known for N = 883)	
Current (%)	97 (11%)
Quit (%)	332 (38%)
Never (%)	454 (51%)
Alcohol Use (known for N = 764)	
Yes (%)	271 (35%)
No (%)	493 (65%)
Any steatosis on liver pathology (%)	718 (67%)
Fibrosis (%)	253 (24%)
Cirrhosis (%)	24 (2%)
Laboratory results	
Complete blood counts (known for N = 1038)	
WBC (K/uL), Median [Q1, Q3]	7.8 [6.5, 9.4]
RBC (M/uL), Median [Q1, Q3]	4.6 [4.3, 4.9]
Hemoglobin (gm/dL), Median [Q1, Q3]	13.7 [13.0, 14.5]
HCT (%), Median [Q1, Q3]	40.0 [37.9, 42.3]
MCV (fL), Median [Q1, Q3]	87.3[84.3, 90.0]
MCH (pg/cell), Median [Q1, Q3]	30.0[28.8, 31.0]
MCHC (gm/dL), Median [Q1, Q3]	34.3 [33.9, 34.7]
Platelet count (K/uL), Median [Q1, Q3]	284 [245, 331]
Folic acid (ng/dL), Median [Q1, Q3]	14.9 [10.9, 20.0]
Serum zinc (ug/L), Median [Q1, Q3]	742 [667, 819]

Of the 1064 patients in our study, 154 of those patients (14%) had some kind of a complication (Table 4). The laparoscopic group had a 9% overall complication rate in comparison to the open group which had an 18% complication rate (Chi-square p < 0.0001). There was no significant difference in the major complication rates between the laparoscopic (n = 29/640, 4.5%) and open (n = 18/424, (4.2%) groups (Chi-square p = .082). Wound infections were the primary complication in both the open and laparoscopic groups, but the rate of both major and minor wound complications as a percentage of complications was higher in the open RYGB cohort (72% open versus 27% laparoscopic, Chi-square p < 0.0001). There were no deaths in this series.

**Table 4:** Summary of 30-day complications for open and laparoscopic RYGB procedures.

	Total N = 1064	Open surgery N = 640		Laparoscopic surgery N = 424	
No complications	910 (86%)	523 (82%)		387 (91%)	
Any complication (major or minor)	154 (14%)	117 (18%)		37 (9%)	
		Major	Minor	Major	Minor
Type of complication*		29	88	18	19
Wound	94	12	72	2	8
Blood loss/anemia	11	7	0	4	0
Respiratory	8	2	5	0	1
Leak	7	1	0	6	0
Stricture	6	0	1	5	0
Nausea/Vomiting/Dehydration	6	1	4	0	1
Renal	5	0	3	0	2
Death	0	0	0	0	0
Other	20	7	5	1	7

*Subjects may have more than one type of complication. Hence, the total number of complications is more than the number of subjects with a complication.

Post-operative surgical complications were then analyzed as a function of iron staining of liver tissue obtained during surgery and pre-operative levels of several serum iron parameters (Table 5). There was no significant difference in any complication, major or minor, between those patients with hepatic iron staining and those with no evidence of increased liver iron. Patients with any complication did have significantly higher pre-operative median serum iron and transferrin saturation levels, while those with major complications also had significantly higher pre-operative median serum iron and transferrin saturation levels, as well as higher serum ferritin. Of those patients with an abnormally elevated serum level of an iron parameter, only abnormally high transferrin saturation levels were associated with significantly more complications of any kind (p = 0.003) and with minor complications (p = 0.001). Major complications were not significantly associated with abnormally elevated levels of any of the serum iron parameters.

**Table 5:** Comparison of iron status with complications.

COMPLICATIONS	None N = 910	Any* N = 154	Any vs. None p-value	Major* N = 47	Major vs. None p-value	Minor* N = 107	Minor vs. None p-value
Hepatic Iron Staining	152 (17%)	31 (20%)	0.30^2^	10 (21%)	0.41^2^	21 (20%)	0.42^2^
Serum iron (ug/dL)							
Median [Q1, Q3]	61 [49, 77]	67 [54, 83]	0.008^1^	69 [57, 83]	0.013^1^	66 [52, 83]	0.097^1^
Abnormal (% > 160)	3 (<1%)	2 (1%)	0.17^2^	0 (0%)	0.99^2^	2 (2%)	0.098^2^
Ferritin (ng/mL)							
Median [Q1, Q3]	99 [49, 163]	105 [62, 195]	0.093^1^	120 [70, 235]	0.024^1^	91 [61, 184]	0.52^1^
Abnormal (% > 400)	30 (4%)	5 (4%)	0.99^2^	4 (10%)	0.083^2^	1 (1%)	0.24^2^
Iron binding capacity (ug/dL)							
Median [Q1, Q3]	316 [288, 348]	316 [284, 352]	0.91^1^	313 [283, 348]	0.77^1^	321 [284, 353]	0.74^1^
Abnormal (% > 428)	21 (3%)	3 (2%)	0.99^2^	1 (3%)	0.99^2^	2 (2%)	0.99^2^
Transferrin saturation (%)							
Median [Q1, Q3]	19 [15, 25]	21 [17, 27]	0.017^1^	24 [19, 28]	0.011^1^	21 [16, 27]	0.19^1^
Abnormal (% > 55)	0 (0%)	3 (2%)	0.003^2^	0 (0%)	NA	3 (3%)	0.0014^2^

*Complications were limited to those occurring within 30-days of discharge.

^1^Wilcoxon rank-sum test; ^2^Fisher's exact test

We then evaluated surgical complications as a function of pre-operative serum iron levels and the hepatic iron overload genetic mutations C282Y and H63D (Table 6). Neither the C282Y nor the H63D mutations were significantly predictive of any complication, or major or minor complications. However, the combination of two allele hits (heterozygous for both C282Y and H63D or homozygous for either) did significantly predict any complication (p = .043).

**Table 6:** Comparison of HFE gene status with complications using Fisher's exact test.

Complications	None N = 910	Any* N = 154	Any vs. None p-value	Major* N = 47	Major vs. None p-value	Minor* N = 107	Minor vs. None p-value
C282Y							
Normal/Hetero, N (%)	907 (99%)	154 (100%)	0.99	47 (100%)	0.99	107 (0%)	0.99
Homo, N (%)	3 (<1%)	0 (0%)		0 (0%)		0 (0%)	
H63D							
Normal/Hetero, N (%)	888 (98%)	146 (95%)	0.065	44 (94%)	0.12	102 (95%)	0.19
Homo, N (%)	22 (2%)	8 (5%)		3 (6%)		5 (5%)	
C282Y/H63D**							
Allele dose <2	870 (96%)	141 (92%)	0.043	43 (91%)	0.27	98 (92%)	0.089
Allele dose 2+	40 (4%)	13 (8%)		4 (9%)		9 (8%)	

*Complications were limited to those occurring within 30-days of discharge

**An allele dose of 2+ includes all patients that were Homozygous for C282Y, Homozygous for H63D, or Heterozygous for both C282Y and H63D.

Because the majority of complications in open RYGB are wound complications, we analyzed iron status in this subset of patients (Table 7). There was no significant difference in the patients with and without wound complications for each HFE mutation, but the patients with at least 2 HFE mutations had a significantly increased risk for wound complications after open RYGB (p = 0.010). Patients with wound complications also had higher pre-operative transferrin saturation levels (p = 0.0027).

**Table 7:** Summary of 30-day wound complications in patients undergoing OPEN RYGB

Wound Complications	None N = 556	Any N = 84	Any vs. None p-value
Hepatic Iron Staining	109 (20%)	14 (17%)	0.66^2^
Serum iron (ug/dL)*			
Median [Q1, Q3]	59 [47, 75]	62 [52, 81]	0.18^1^
Abnormal (% > 55)	2 (<1%)	2 (3%)	0.097^2^
Ferritin (ng/mL)*			
Median [Q1, Q3]	103 [55, 167]	94 [62, 184]	0.89^1^
Abnormal (% > 55)	21 (4%)	2 (3%)	0.76^2^
Iron binding capacity (ug/dL)*			
Median [Q1, Q3]	314 [288, 347]	323 [284, 353]	0.73^1^
Abnormal (% > 55)	13 (3%)	2 (3%)	0.99^2^
Transferrin saturation (%)*			
Median [Q1, Q3]	19 [15, 25]	21 [16, 26]	0.33^1^
Abnormal (% > 55)	0 (0%)	3 (4%)	0.0027^2^
C282Y			
Normal/Hetero, N (%)	555 (99%)	84 (100%)	0.99^1^
Homo, N (%)	1 (<1%)	0 (0%)	
H63D			
Normal/Hetero, N (%)	543 (98%)	79 (94%)	0.074^1^
Homo, N (%)	13 (2%)	5 (6%)	
C282Y/H63D**			
Allele dose <2	535 (96%)	75 (89%)	0.010^1^
Allele dose 2+	21 (4%)	9 (11%)	

*Serum iron, ferritin, iron binding capacity, and transferring saturation were available for n = 541, n = 546, n = 542, and n = 540 subjects, respectively.

**An allele dose of 2+ includes all that were homozygous for C282Y, homozygous for H63D, and compound heterozygous for both C282Y and H63D.

^1 ^Chi-square ^2 ^Fisher's Exact test

Complications can affect the post-operative length of stay. The mean length of stay (LOS) was 2.6 days, which was not significantly different by hepatic iron staining or by serum iron parameters (data not shown). We also evaluated those patients with prolonged LOS (>4 days). Transferrin saturation and ferritin levels were higher in patients with LOS > 4 days (Table 8). No significant difference in LOS was found based on HFE gene mutation status (data not shown).

**Table 8:** Comparison of iron status with length of stay.

Length of stay	<4 days N = 947	4+ days N = 117	p-value
Hepatic Iron Staining	159 (17%)	24 (21%)	0.30^2^
Serum iron (ug/dL)*			
Median [Q1, Q3]	61 [49, 77]	63 [52, 83]	0.062^1^
Abnormal (% > 160)	5 (<1%)	0 (0%)	0.99^2^
Ferritin (ng/mL)*			
Median [Q1, Q3]	94 [49, 163]	118 [68, 195]	0.0093^1^
Abnormal (% > 400)	33 (4%)	2 (2%)	0.42^2^
Iron binding capacity (ug/dL)*			
Median [Q1, Q3]	316 [287, 348]	313 [290, 352]	0.68^1^
Abnormal (% > 428)	22 (3%)	2 (2%)	0.99^2^
Transferrin saturation (%)*			
Median [Q1, Q3]	19 [15, 25]	22 [17, 27]	0.026^1^
Abnormal (% > 55)	3 (<1%)	0 (0%)	0.99^2^

*Serum iron, ferritin, iron binding capacity, and transferring saturation were available for n = 894, n = 900, n = 895, and n = 891 subjects, respectively.

^1^Wilcoxon rank-sum test; ^2^Fisher's exact test

We then reanalyzed the data in the context of gender, the presence of type 2 diabetes, and hypertension (data not shown). Except for open versus laparoscopic comparisons, most of the results were not impacted by stratification either by gender, diabetes, or hypertension, except for a higher rate in minor complications in females (10% vs. 5%, p < 0.035) and in any complications in non-diabetics (8% vs. 2%, p < 0.013) in patients with the combination of two HFE allele hits (heterozygous for both C282Y and H63D or homozygous for either). Re-analysis of data for 30-day complications in open versus laparoscopic RYGB procedures after stratifying for gender, the presence of type 2 diabetes and hypertension (data not shown), revealed a statistically higher percentage of females with abnormal serum iron levels (3% vs. <1%, p < 0.049) and a higher percentage of females who were homozygous for the HFE H63D allele (8% vs. 2%, p < 0.032) for those with any complication. Iron binding capacity in males was higher (335 vs. 304, p < 0.049) in those with wound complications. More non-diabetics with wound complications were homozygous for the H63D allele (11% vs. 1%, p < 0.0049).

The relationship of serum iron parameters and hepatic iron staining to HFE gene status was also determined (Table 9). No association was found with either the C282Y or H63D mutations by themselves, in part due to the reduced power from small numbers of homozygotes. However, increased serum iron (p < 0.0005), transferrin saturation (p < 0.0001), and percentage with elevated ferritin levels (p < 0.037) was found in patients with 2 or more HFE mutations, whether C282Y or H63D, consistent with their expected effects on iron metabolism.

**Table 9:** Comparison of HFE gene status with iron status.

	Hepatic Iron Staining		Serum Iron (ug/dL)			Ferritin (ng/mL)			Iron binding capacity (ug/dL)			Transferrin saturation (%)		
	**N with data**	**Iron staining (%)**	**N with data**	**Median [Q1, Q3]**	**Abnormal (% > 160)**	**N with data**	**Median [Q1, Q3]**	**Abnormal (% > 400)**	**N with data**	**Median [Q1, Q3]**	**Abnormal (% > 428)**	**N with data**	**Median [Q1, Q3]**	**Abnormal (% > 55)**

C282Y														
Normal/Hetero (N = 1061)	1061	182 (17%)	892	62 [50, 79]	5 (<1%)	898	99 [50, 167]	34 (4%)	893	316 [287, 349]	24 (3%)	889	20 [15, 25]	3 (<1%)
Homo (N = 3)	3	1 (33%)	2	106 [106, 106]	0 (0%)	2	306 [112, 500]	1 (50%)	2	316 [284, 349]	0 (0%)	2	34 [30, 37]	0 (0%)
p-value ^A^		0.43		NA	0.99		NA	0.076		NA	0.99		NA	0.99
H63D														
Normal/Hetero (N = 1034)	1034	178 (17%)	868	62 [49, 79]	5 (<1%)	872	99 [50, 167]	33 (4%)	869	316 [287, 349]	23 (3%)	865	20 [15, 25]	2 (<1%)
Homo (N = 30)	30	5 (17%)	26	63 [57, 67]	0 (0%)	28	86 [44, 171]	2 (7%)	26	316 [272, 348]	1 (4%)	26	21 [17, 24]	1 (33%)
p-value ^A^		0.99		0.75	0.99		0.75	0.30		0.38	0.51		0.49	0.085
C282Y/H63D*														
Allele dose <2 (N = 1011)	1011	172 (17%)	847	61 [49, 78]	3 (<1%)	851	99 [50, 167]	30 (4%)	848	317 [288, 349]	23 (3%)	844	19 [15, 25]	0 (0%)
Allele dose 2 + (N = 53)	53	11 (21%)	47	69 [59, 90]	2 (4%)	49	91 [49, 187]	5 (10%)	47	298 [272, 335]	1 (2%)	47	24 [19, 32]	3 (6%)
p-value ^A^		0.46		0.0005	0.024		0.98	0.037		0.0073	0.99		<.0001	0.0001

*An allele dose of 2+ includes all patients that were Homozygous for C282Y, Homozygous for H63D, or Heterozygous for both C282Y and H63D.

A = p-values are resulted from Fisher's exact test and wilcoxon rank sum test

## Discussion

Minimizing peri-operative complications in RYGB is important because morbidly obese patients have significant operative risks, many related to associated co-morbidities. Iron status is a potentially important factor influencing these co-morbidities. We found that increased serum iron and transferrin saturation measured pre-operatively were associated with post-operative complications and that increased serum ferritin, as well as iron and transferrin saturation, were associated with major complications. Pre-operatively increased serum transferrin saturation was also associated with wound complications following open RYGB and transferrin saturation and ferritin with prolonged lengths of stay. The presence of 2 or more HFE mutations was associated with overall complications as well as wound complications in open RYGB. No differences were found in complication rates between those with stainable liver iron and those without histological evidence of hepatic iron overload. Stratifying by gender and diabetes also revealed several other associations of higher iron status in patients with complications.

The relationship between iron status and complications may be due oxidative stress. Iron is the most abundant transition metal in the human body and is an essential element for life [20]. It is potentially toxic because of its ability to generate toxic free radicals and resulting cellular injury from oxidative stress [21]. As a protective measure, iron is sequestered in proteins, particularly ferritin and transferrin. Iron-catalyzed generation of oxidative stress has been implicated in many clinical disorders, including wound healing and infection. In wounds there is a low pH, a partial ischemic state, and an excess of free radicals [22]. Under these conditions, iron may be released from storage, thereby making it available to catalyze further tissue damage. Our findings that serum iron and transferrin saturation were increased in patients with complications, particularly wound complications, is consistent with the availability of increased iron that could play a role in the pathogenesis of complications. We found only a higher level of ferritin in patients with major complications. Ferritin is also an acute phase response protein and may be a marker for a sub-clinical inflammatory state pre-disposing to major complications in open RYGB and not reflecting differences in iron status.

Iron may also be deleterious if present in insufficient amounts. It is required for the hydroxylation of proline and lysine required in collagen synthesis, thus iron deficiency may collagen production and delay wound healing [23]. A reduction in oxygen carrying capacity with iron deficiency may also affect wound healing. Iron levels need to be in an optimal range, neither deficiency not elevated, in order for optimum physiological responses to surgical procedures.

Consistent with the results for serum iron parameters, we found that patients carrying 2 or more HFE gene mutations had a two-fold higher rate of overall complications, though each individual mutation did not reach statistical significance. The HFE C282Y mutation has been associated with venous leg ulceration [24] suggesting that HFE genotype may affect wound complications and healing. Patients with HFE gene mutations absorb increased amounts of dietary iron that is characteristically reflected as an elevation of transferrin saturation [25]. The HFE C282Y and H63D mutations cause increased iron absorption whether in a homozygous, i.e., C282Y/C282Y or H62D/H62D, or compound heterozygous configuration, i.e., C282Y/H63D. However, the biochemical expression of HFE mutations is variable. In a study of over 200 C282Y homozygotes, a total of 28% of male C282Y homozygotes had evidence of iron-overload-related disease [26]. In contrast, the prevalence of iron-overload-related disease was only 1% in female C282Y homozygotes, which may be due to a lower iron burden and/or other modifying effects. In our population, about 80% of the patients were female, thus the clinical expression of mutations may be under-represented.

Despite the association of complications with serum iron indices, the presence of stainable iron in the liver was not associated with post-operative RYGB complications. The liver serves as a major storage depot for total body iron. Total body iron stores are generally proportional to serum ferritin, although the correlation is not very strong [27]. In the absence of HFE mutations, the levels of serum iron and transferrin saturation may not be indicative of increased iron absorption and increased iron stores, thus stainable liver iron may not be expected to predict the same outcomes as serum iron and transferrin saturation.

A higher rate of wound complications, defined as superficial and deep surgical site infections and dehiscence, was found in patients undergoing open RYGB versus those who had laparoscopic RYGB. This is similar to reports in the literature of wound problems occurring in about 10-20% of patients following open RYGB [19,28]. A majority of the complications in RYGB were considered minor, whereas in laparoscopic RYGB approximately equal numbers of major and minor complications were seen.

Obesity is a known risk factor for several types of complications in other surgeries. For example, overall postoperative complications have been associated with BMI in patients with non-perforated appendicitis [29]. Increased BMI has also been associated with increased wound complications in both minimally invasive and open rectal surgery [30,31]. Percent body fat calculated by bioelectrical impedance analysis has been associated with a 5-fold increased risk of surgical site infections after elective surgery [32]. However, BMI may not affect complication rates for all types of operations. Although total laparoscopic hysterectomy for obese patients required significantly longer to complete and was associated with a higher risk of significant blood loss, major and minor complications, hospital readmission, and reoperation were not different [33].

There are several limitations to our study. The only operation studied was RYGB. The findings do not apply to other types of bariatric surgeries, such as the laparoscopic band procedure. The relative number of men enrolled in the study was much lower than the number of women, so limited statistical power exists to determine whether the associations found for female patients are truly not present in males. Follow-up studies with larger numbers of male patients will be required. The numbers of C282Y and H63D homozygotes was also relatively low and thus not sufficiently powered to determine whether these genotypes were associated with differences in complication rates. A much larger sample size will be required given the low rates of these mutations. Our population also consisted of only Caucasian patients. Whether iron status in patients of other races/ethnicities is associated with post-RYGB complications will need to be determined in other studies. We also did not address independent effects of other variables on complications, such as blood glucose levels and BMI.

## Conclusion

In summary, serum markers for iron and HFE gene mutations were associated with complications after RYGB. The serum iron indices and HFE gene mutations may be markers for patient at risk for complications and wound healing problems. The use of genetic markers to identify patients at risk for surgical outcomes, i.e., "surgicogenomics" [14] is analogous to using genetic variants to predict response to medications, i.e., pharmacogenomics. The precise role of iron and HFE mutations in the pathophysiology of post-operative complications in morbidly obese patients following RYGB needs to be further defined.

## Competing interests

The authors declare that they have no competing interests.

## Authors' contributions

GSG and ATP were responsible for developing the idea of this study. GSG, ATP, CDS, and PB contributed to the study design. RC and ATP were involved in the records review and data acquisition. CGW conducted the statistical analyses. GSG performed the literature review and drafting of the manuscript. All authors were involved in reviewing and editing the manuscript. All authors read and have approved the final manuscript.
